# Prognostic Value of Facial Nerve Antidromic Evoked Potentials in Bell Palsy: A Preliminary Study

**DOI:** 10.1155/2012/960469

**Published:** 2011-11-23

**Authors:** Zhang WenHao, Chen Minjie, Yang Chi, Zhang Weijie

**Affiliations:** Ninth People's Hospital, Shanghai Jiao Tong University School of Medicine, Shanghai 200011, China

## Abstract

To analyze the value of facial nerve antidromic evoked potentials (FNAEPs) in predicting recovery from Bell palsy. *Study Design*. Retrospective study using electrodiagnostic data and medical chart review. *Methods*. A series of 46 patients with unilateral Bell palsy treated were included. According to taste test, 26 cases were associated with taste disorder (Group 1) and 20 cases were not (Group 2). Facial function was established clinically by the Stennert system after monthly follow-up. The result was evaluated with clinical recovery rate (CRR) and FNAEP. FNAEPs were recorded at the posterior wall of the external auditory meatus of both sides. *Results*. Mean CRR of Group 1 and Group 2 was 61.63% and 75.50%. We discovered a statistical difference between two groups and also in the amplitude difference (AD) of FNAEP. Mean *± *SD of AD was *−*6.96% *± *12.66% in patients with excellent result, *−*27.67% *± *27.70% with good result, and *−*66.05% *± *31.76% with poor result. *Conclusions*. FNAEP should be monitored in patients with intratemporal facial palsy at the early stage. FNAEP at posterior wall of external auditory meatus was sensitive to detect signs of taste disorder. There was close relativity between FNAEPs and facial nerve recovery.

## 1. Introduction

Bell palsy is a neuropathy of the peripheral seventh cranial nerve, usually resulting from traumatic, compressive, infective, inflammatory, or metabolic abnormalities. However, in many cases no etiology is identified, and the eventual diagnosis is idiopathic [1]. Its annual incidence was from 0.011% to 0.040% [2]. For many patients, the questions that whether their facial function will return to normal one day and how long this is going to take are mostly concerned about. Evaluation of the prognosis of Bell palsy is useful for counseling of patients and guiding further management. Since 1970s, prognostication has been based mainly on various electrophysiologic tests [3–10], such as electromyography (EMG), electroneurography (ENoG), maximal nerve excitability testing, and facial motor nerve conduction (MNC) testing. However, the electrophysiologic tests above are facial nerve orthodromic evoked potentials. Abnormal findings from these tests are obtained after the degeneration-process extends to the extratemporal segment of the facial nerve with 1- to 2-week delay [11]. Decompression surgery cannot play a part in retrieve and prevent degeneration after most of facial nerve function has already degenerated. Therefore, if we want to detect nerve degeneration and to predict facial function recovery during its early stages, it is necessary to use a test that can diagnose degeneration within 1 week after the onset of paralysis.

The facial nerve antidromic evoked potentials (FNAEP) was first described by Bumm et al. in 1974 [12]. It is the only one method to represent the intratemporal facial nerve function [13]. It has the possibility to diagnose nerve degeneration during the early stage of paralysis. Nakatani et al. [14] had used FNAEP to value the prognosis of facial paralysis. However, the correlativity of intra- or extra-temporal facial nerve was not pointed out, and the quantitative analysis was not obtained. The purpose of this study was to evaluate the prognostic use of FNAEP quantitatively within three days in a successive series of patients with Bell palsy associated with or without taste disorder at a university-based center. 

## 2. Patients and Methods

### 2.1. Populations

From January to December 2010, there were 46 patients with unilateral Bell palsy in a single center (Department of Oral and Maxillofacial Surgery, Ninth People's Hospital, Shanghai Jiao Tong University School of Medicine). Out of the patients, 23 were male and 23 were female. Ages ranged from 19 to 66 years (mean, 43.5 yrs). The duration from onset to treatment was from 1 to 3 days (mean, 2.9 d). The right side was involved in 24 patients, and the left was in 22 patients. The etiology was idiopathic. The patients were divided into two groups: 26 patients with taste disorder (Group 1) and 20 patients without taste disorder (Group 2). Interventions given to the patients were local physical therapy and pharmacotherapy, such as high-dose prednisone within 3 days after onset, besides methylcobalamin and vasodilators in 1 month after onset. Forty-three patients (93.5%) were followed up with the average follow-up period of 4.8 months (ranged from 1 to 9 months).

### 2.2. Clinical Evaluation of Facial Nerve Function

The initial and final facial nerve function was reported using the Stennert system [15] (Table 1). Each indicator was scored 10. The score ranged from 0 to 200. Clinical recovery rate (CRR) = (200 − follow-up score)/200 × 100%. The recovery outcomes of facial nerve function was graded as follow: excellent (CRR ≥80%), good (50% ≤ CRR < 80%), and poor (CRR <50%). 

### 2.3. FNAEP Evaluation of the Facial Nerve Function

**Figure 1:**
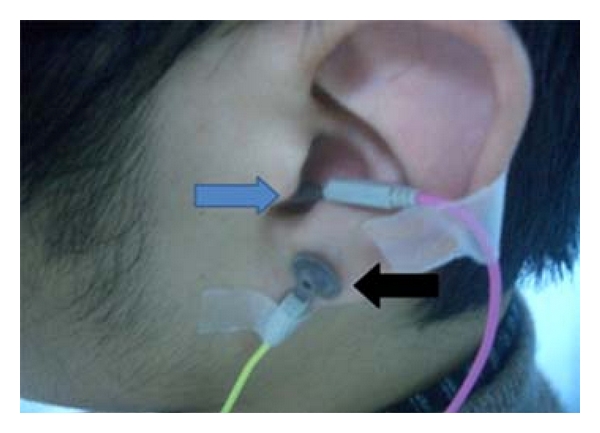
The recording electrode of the posterior wall of the external auditory canal (blue arrow) and the reference electrode of posterior wall of the earlobe (black arrow).

**Figure 2:**
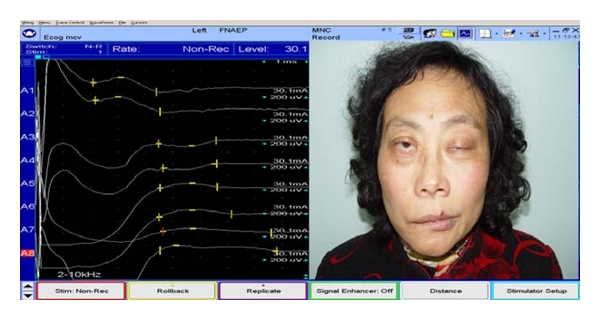
The waveform of FNAEP. A1–A4 show the recorders of asympotomatic side; A5–A8, which amplitude decreased (arrow), show the recorders of sympotomatic side.

The FNAEP device (Viking Quset, Nicolet Corp, USA) includes bipolar stimulators, discoid electrodes, needle electrodes, and monitor. The patients were examined by FNAEP for the first presentation at the clinic. Before the test, the cerumen of external auditory canal was cleared and the degrease cream was embrocated at the external auditory canal and the earlobe. Ground wire was connected on a wrist. Two discoid electrodes with a little conductive paste were located at the posterior wall of the external auditory canal (recording electrode) and the earlobe (reference electrode), respectively (Figure 1). FNAEP was performed first on the asymptomatic side and then repeated on the symptomatic side. The superficial projection of the homolateral stylomastoid foramen was stimulated by the bipolar stimulator with band-pass filtering of 2–10000 Hz and stimulus intensity of 30 mA. The results of both sides were recorded (Figure 2). The amplitude difference (AD) and latency difference (LD) between symptomatic side and asymptomatic side were calculated according to the following formula:

(1) $$\begin{array}{c} \text{AD}=\frac{\left(\text{amplitude}\;\text{of}\;\text{symptomatic}\;\text{side}-\text{amplitude}\;\text{of}\;\text{asymptomatic}\;\text{side}\right)}{\text{amplitude of}\;\text{asymptomatic}\;\text{side}}\times 100\%,\\ \text{LD}=\;\frac{\left(\text{latency}\;\text{of}\;\text{symptomatic}\;\text{side}-\text{latency}\;\text{of}\;\text{asymptomatic}\;\text{side}\right)}{\text{latency}\;\text{of}\;\text{asymptomatic}\;\text{side}}\times 100\text{\%}. \end{array}$$

### 2.4. Statistical Analysis

Statistical analysis of the data, presented as means ± SD, was performed using SAS 8.1 software. The difference between “Group 1” and “Group 2” was analyzed. Significance was established when probability was *P* < 0.01. Statistical difference was established when probability was 0.01 <*P* < 0.05. No statistical difference was established when probability was *P* > 0.05.

## 3. Results 

### 3.1. Clinical Facial Nerve Function

**Figure 3:**
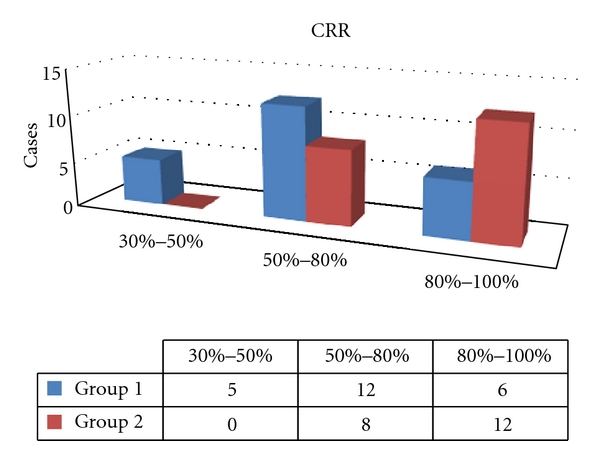
The CRR in the group with taste disorder and the group without taste disorder.

During the period of followup, the mean CRR was 61.63%  ± 18.90% (ranged from 30% to 90%) in Group 1. There were 6 cases (26.09%) with excellent result, 12 cases (52.13%) with good result, and 5 cases (21.74%) with poor result. In Group 2, the mean CRR was 75.50% (ranged from 50% to 100%). There were 12(60%) cases with excellent result, 8 cases (40%) with good result, and no cases with poor result (Figure 3). There was a statistical difference between the two groups (*P* = 0.0189 < 0.05). 

### 3.2. FNAEP Results

**Figure 4:**
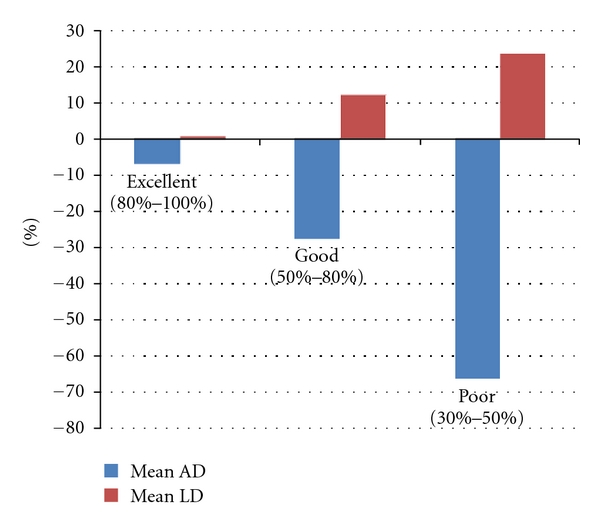
Mean AD and LD in the groups with excellent, good, and poor clinical results.

The mean of AD of Group 1 and Group 2 were −33.88% and 5.47%. There was a significant difference between them (*P* < 0.01). For the patients with excellent result, mean ± SD of AD and LD were −6.96%  ± 12.66% and 0.54%  ± 7.23%. For the patients with good result, mean ± SD of AD and LD were −27.67%  ± 27.70% and 12.11%  ± 7.23%. For the patients with poor result, mean ± SD of AD and LD were −66.05%  ± 31.76% and 23.36%  ± 1.61% (Figure 4). There was a significant difference of AD between the “excellent” group and the “poor” group (*P* = 0.0014 < 0.01), and there were statistical differences between the “excellent” group and the “good” group (*P* = 0.010 < 0.05) and between the “good” group and the “poor” group (*P* = 0.0287 < 0.05). There was a statistical difference of LD between the “excellent” group and the “poor” group (*P* = 0.0124 < 0.05), and there was a significant difference between the “excellent” group and the “good” group (*P* = 0.0041 < 0.01) and no significant difference between the “good” group and the “poor” group (*P* = 0.2041 > 0.05). 

## 4. Discussion

The majority of the drug treatments for Bell palsy at the early stage are effective. Nevertheless, those patients who are not completely improved by medication deserved facial nerve decompression surgery. Accordingly, for almost all the patients, the questions that whether their facial function will return to normal and the facial nerve decompression surgery is necessary or not and when to take are mostly concerned about. Evaluation of the prognosis of Bell palsy is useful for counseling of patients and guiding further management. Although electrical tests were already introduced to predict the prognosis of Bell palsy in the 1970s, they were still controversial. As the facial nerve is stimulated out of the temporal bone in these tests, the evaluation of nerve function is limited to the extratemporal facial nerve. There is general agreement that abnormal findings from these tests are obtained after the degeneration process extends to the extratemporal segment of the facial nerve with 1-week delay [3–10]. We are not able to obtain information about facial nerve damage in the temporal bone at the early stage of facial palsy with extratemporal electrodiagnostic tests, such as MNC, EMG, and ENoG. Decompression surgery cannot play a part in retrieve and prevent degeneration after most of the nerve function has already degenerated. To raise therapeutic effect, we should precisely evaluate the poor outcome that patients who suffered from Bell palsy are possible to gain before completion of facial nerve degeneration. With regards to this, it is necessary to use a test that can diagnose degeneration within 1 week after the onset of paralysis.

The FNAEP is the only method of monitoring a nerve action potential, among all the electrodiagnostic tests of the facial nerve [3–10]. Other than traditional electrophysiological testing, FNAEP stimulated the extratemporal segment of facial nerve and was recorded at the intratemporal segment. If the lesion occurred, its amplitude would cause abnormal changes, which provided strong evidence of intratemporal location [16]. The waveform of this potential reaction was more constant and had obvious time locked relationship with the stimulus. Tashima et al. [17] reported that the recorder located at the posterior wall of external auditory meatus could represent the vertical portion of facial nerve, and the characteristic waveform of the FNAEP was also revealed in animal experiments. 

Taste disorder in facial paralysis implies the lesion of vertical portion of facial nerve intratemporally. In this study, the duration from onset to test was from 1 to 3 days, and the mean of AD of Group 1 and Group 2 were −33.88% and 5.47%. There was a significant difference between them. Also, there was a statistical difference of CRR between two groups. Significant abnormal of AD of FNAEP was correlative with taste disorder. It was confirmed that FNAEP can prompt facial nerve damage in the temporal bone, and recorder at the posterior wall of external auditory meatus was appropriate. With the preliminary assessment, we found that the relativity between FNAEP amplitude and the clinical results was closer than that between latency and the clinical results, and the symptom of taste disorder would occur if AD of FNAEP was lower than −30%. It was important to evaluate the nerve function of intratemporal segment at the early stage. FNAEP was the best choice to evaluate and predict the facial nerve function at the early stage. 

Herzon et al. [18] never applied FNAEP to evaluate prognosis of facial paralysis. Comparison of FNAEP between symptomatic side and asymptomatic side showed that the clinical results were good when only amplitude was temporarily discrete, and the results were poor while amplitude obviously reduced. However, Herzon failed to obtain quantitative analysis. According to our quantitative analysis, it was preliminary considered that when AD between symptomatic side and asymptomatic side was ranged from 0 to −20%, the predicted result was excellent; when it was ranged from −20% to −50%, the predicted result was good and when it was ranged from −50% to −100%, the predicted result was poor; facial nerve decompression may be considered.

## Figures and Tables

**Table 1:** Stennert system.

Facial paralysis symptoms	Evaluating standard	Secondary damage	Evaluating standard
Static state		Hyperacusis	Yes
Bilateral palpebral fissure difference	≥3 mm	Taste disorder	Yes
Lower eyelid ectropion	Yes	Joint movement: Amount, eyes, nasolabial fold, mouth and cheek
Nasolabial fold loss	Yes	
Ptosis of labial angle	≥3 mm	Two among the above	Yes
Movement		Three among the above	Yes

Frown	No	Blink (secondary spasm)	Yes
Palpebral fissure could not close:		Contracture	Yes
Sleeping	Yes	Tear secretion:	
Maximum stimulation	Yes	Palpebral fissure static ≥70%	Yes
Grin: upper and lower canines	Not visible	<70%	Yes
Upper lateral incisors	Not visible	0%	Yes
Whistle: distance between philtrum and mouth corner on diseased side more than that on healthy side	50%	Tear	Yes
	

Acial paralysis index		Secondary damage index	
